# Ecology of West Nile Virus in North America

**DOI:** 10.3390/v5092079

**Published:** 2013-09-04

**Authors:** William K. Reisen

**Affiliations:** Center for Vectorborne Diseases and Department of Pathology, Microbiology and Immunology, School of Veterinary Medicine, University of California Davis, CA 95616, USA; E-Mail: wkreisen@ucdavis.edu; Tel.:530-752-0124; Fax 530-754-6086

**Keywords:** West Nile virus, *Culex*, ecology, North America, invasion, persistence

## Abstract

The introduction, dispersal and establishment of West Nile virus in North America were reviewed, focusing on factors that may have enhanced receptivity and enabled the invasion process. The overwintering persistence of this tropical virus within temperate latitudes was unexpected, but was key in the transition from invasion to endemic establishment. The cascade of temporal events allowing sporadic amplification to outbreak levels was discussed within a future perspective.

## 1. Introduction

West Nile virus (WNV) was discovered originally in 1937 during a fever survey in the West Nile district of Uganda [1] and has been classified immunologically within the Japanese encephalitis serocomplex in the genus *Flavivirus,* along with Japanese encephalitis in Asia, St. Louis encephalitis (SLEV) in the New World and Murray Valley in Australia. Historically, its distribution was limited to Africa and Asia, with occasional intrusions into southern Europe, possibly by migratory birds [2]. The subsequent arrival of this tropical African virus into the sophisticated concrete jungle of New York City (NYC) in 1999 was totally unexpected and immediately captured both media as well as scientific attention. The resulting public, veterinary and wildlife health impacts were unprecedented and brought together widely disparate groups such as the Nature Conservancy and the American Mosquito Control Association to discuss insecticide applications for intervention. New research and public health programs supported, in part, by Epidemiology and Laboratory Capacity funding from the US Centers for Disease Control and Prevention (CDC), expanded surveillance, testing and reporting programs that tracked the rapid invasion of the continental United States (http://www.cdc.gov/ncidod/dvbid/westnile/index.htm). The resulting volume of research on WNV has been staggering and has exploited the recent proliferation of online health and other journals, as evidenced from a March 2013 search for PubMed titles containing ‘West Nile’ that returned 3033 results. This recent extensive data and associated literature has generated a series of excellent reviews that have summarized the virus in general [3,4,5,6,7,8,9,10,11], pathogenesis in human [12,13,14,15,16,17,18], equine [19] and avian [20] hosts, epidemiological patterns [21,22], ecology [23,24,25,26], dispersal [27,28,29,30,31], impact on avifauna [32,33,34,35], and mosquito bionomics [25,36], experimental vector competence [37,38] and blood feeding patterns [39]. 

The current review addresses aspects of the ecology and epidemiology of WNV that have received somewhat less attention, although redundancy of thought and content will be inevitable. The resulting synthesis benefited from discussions during recent meetings at the American Academy of Microbiology’s mini colloquium “FAQ: West Nile Virus” and at the US National Institute of Environmental Health Sciences meeting “Extreme Weather, Climate and Health: Putting Science into Practice”. My approach will focus first on how anthropogenic change in North America set the stage for the successful invasion and dispersal of WNV, and then on the importance of early season events including persistence and amplification for the onset of summer outbreaks. 

## 2. Global Distribution

**Figure 1:**
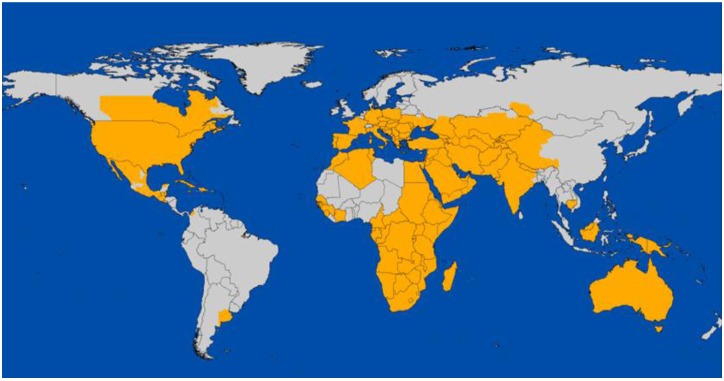
Global distribution of West Nile virus, 2006 [Figure courtesy of the US Centers for Disease Control and Prevention.].

West Nile virus is perhaps the widest distributed arbovirus globally, being now found on all the continents except Antarctica and from tropical to north temperate latitudes (Figure 1). Like many microbial pathogens, the historical distribution and apparent dispersal of WNV may be confounded by resources and methods available for detection. However, since its initial isolation in Uganda, WNV seems to have spread and/or been initially reported from outbreaks throughout Africa in the 1950s and 1970s, India during the 1950s, the Mediterranean region and Eastern Europe during the 1990s [2,11], and finally the New World in the 2000s [4,40,41]. A distinct grouping in lineage 1b, known as Kunjin virus, has been reported in Australia since first isolated in 1960 [42]. In developing countries such as Pakistan where WNV appears to be endemic, human disease seems lost among the myriad of childhood febrile illnesses and the annual cohort seroconversion rate typically is progressive and consistent [43], with older individuals protected via acquired immunity. In contrast, ‘virgin soil’ transmission such as seen in eastern Europe, Greece and the United States has produced clinically severe neuroinvasive disease in non-immune older age groups [14], similar to that seen with SLEV during novel outbreaks in parts of the United States [44]. 

## 3. Transmission Cycle in the Old World

WNV is an avian zoonosis, being maintained in nature by transmission among ornithophagic *Culex* mosquitoes and a wide-variety of birds, especially those in the order Passeriformes. Several *Culex* species have been implicated as vectors based mostly on laboratory vector competence studies, including *Culex univittatus, Culex neavei* and perhaps the *Culex pipiens* complex in Africa [45,46,47], *Cx. pipiens* complex and perhaps *Culex modestus* and *Culex perexiguus* in Europe [48], *Culex annulirostris* and perhaps the *Cx. pipiens* complex in Australia [49,50], and *Culex bitaeniorhynchus, Culex vishnui, Culex pseudovishnui, Culex tritaeniorhynchus* and *Culex quinquefasciatus* in India and Pakistan [51,52,53]. However, the exact role of these species in virus epidemiology has been confusing due to their frequent blood feeding on large mammals and the limited numbers of isolations made during outbreaks and ecological investigations [54]. A large variety of migratory and resident birds species have been found naturally infected [2,55,56], but few host competence studies have been conducted to ascertain their importance in transmission. In addition, the apparent repeated introduction of WNV into Europe seems to have resulted in minimal avian mortality, despite the fact that several isolates from outbreaks have contained the NS3 T249P mutation associated with virulence in American Crows in North America (NA) [57]. Interestingly, mortality in southbound migrating White Storks (Ciconiidae) was reported in Israel, and this WNV strain later was associated with wide-spread mortality in domestic geese [58] and was closely related to the strain that invaded NYC the following year [40].

## 4. Invasion of North America

### 4.1. Setting the Stage

The colonization of NA by Europeans greatly changed the landscape and markedly increased the size of the human population. Intensive agriculture and the need for construction supplies fragmented the deciduous forests. Points of trade by sailing ships produced cities along the eastern seaboard and then along major waterways. With a large number of humans and animals concentrated into permanent urban settings, waste disposal became a major problem and produced refuse dumps and highly eutrophic municipal water systems for waste and storm run-off. With urbanization came a reduction in avian species diversity [59,60], but an expansion of the number and range of commensal species such as American Robins, House Finches and American Crows that were able to exploit peridomestic habitats. In addition, House Sparrows, European Starlings and Rock Doves were intentionally released into Eastern NA and rapidly exploited the expanding urban environment throughout the continent. This reduction in urban avian diversity [61] left a guild of commensal species, many of which were competent hosts for WNV [62]. The *Culex* vectors of WNV seemed to be opportunistic feeders able to exploit whatever avian or mammalian hosts were abundant in the environment [63,64,65,66,67]. Simplification of avian diversity therefore focused vector blood meal acquisition on a few competent species, facilitating infection and transmission and increasing the efficiency of viral amplification [68,69]. 

The need to carry drinking water for long ocean voyages allowed the unintentional transport and introduction of several mosquito species, including members of the *Culex pipiens* complex that included the Northern and Southern House Mosquitoes, aptly named for their close association with humans. This complex apparently arose within the Ethiopian region [70], but now is distributed circumglobally [71], being able to survive cold northern winters as well as exploit warm southern latitudes, with hybrids found at intervening latitudes [72,73,74]. A third member of the complex, *Cx. pipiens* form molestus seems to have evolved from above ground *Cx. pipiens* populations [75] to exploit underground collections of water in temperate [36], but not tropical latitudes, where these underground habitats are exploited by *Cx. quinquefasciatus*. Females in this complex typically blood feed on birds, but southern and admixed populations also feed on humans and dogs [64,67]. Other rural *Culex* such as *Culex nigripalpus* in the southeast and *Culex tarsalis* in the west have exploited irrigated agricultural landscapes, managed wetlands and some urban habitats. Both species feed on both avian and mammalian hosts, but shift to more frequent mammal feeding during late summer, thereby functioning effectively as both enzootic and bridge vectors [67,76,77].

This mixture of urban birds and peridomestic mosquitoes living in close proximity to humans created situations conducive for the transmission of arboviruses, especially SLEV. Although this virus is apparently endemic to the New World, it was not discovered until 1933, when there was a large outbreak of human disease associated with lower socio-economic housing, poor waste water management, large *Cx. pipiens* populations, and exceptionally hot and dry weather in St Louis Missouri [78], conditions now associated with WNV outbreaks. Subsequently, SLEV was found throughout NA, where it caused extensive epidemics of human neurological disease, especially in the Ohio River Valley during the 1970s [44]. Improved intervention through organized mosquito control and urban waste water management seems to have eliminated large epidemics, leaving most human and avian populations without acquired flavivirus immunity. 

### 4.2. The Invasion

Prior to its discovery in NA, WNV had been a virus on the move, with small outbreaks recorded in the Mediterranean region and epidemics of neuroinvasive disease documented in Romania [79] and Russia [9,80,81,82]. In 1998 there was an outbreak of WNV in Israel, and this virus strain was similar genetically to that introduced into NYC [40]. There is frequent air travel between NYC and Israel, and it was most likely that the virus was introduced by this frequent and repeated route of travel.

Similar to the SLEV outbreak in 1933, multiple factors in NYC during 1999 set the stage for the successful invasion and outbreak of WNV. The decrease in endemic arbovirus activity in prior years resulted in the closing of arbovirus surveillance and most mosquito control programs in the NYC area, except for a small program on Long Island retained to control pestiferous salt marsh mosquitoes. The summer and especially July of 1999 was the hottest in NYC recorded history and was associated with below average rainfall. These weather conditions were conducive for the production of large numbers of *Cx. pipiens* complex mosquitoes from storm water systems partially dammed with debris and enriched with leaves. Warm weather typically speeds larval mosquito development, shortens population generation times and thereby accelerates growth of mosquito populations. In addition, the commensal avian population and most of the human population had no immunity against flaviviruses. 

During the summer of 1999, large numbers of American Crows were observed dead and dying in and around NYC [83], and exotic birds from collections at the Bronx zoo were dying [20]. A virus isolated from a deceased Chilean flamingo grouped with lineage 1 of WNV [40]. Interestingly, the NY99 virus strain, as well as multiple isolates from outbreaks in Europe, carried the T249P mutation in the NS3 region of the viral genome that was associated experimentally with elevated viremias and 100% mortality in American Crows [57], but not necessarily other corvids. Concurrently, a small cluster of neuroinvasive disease cases was recognized and diagnosed serologically, initially as SLEV, and then as WNV. Although the actual mechanism or date of WNV introduction probably will never be known, it seems likely that the virus was introduced by air traffic from Israel. Humans and equines (that also travel frequently by air) are considered to be ‘dead end’ hosts for the virus; however, some *Culex* can infrequently become infected after feeding on fairly low doses of WNV [84]. Alternatively, mosquitoes often are inadvertently transported on aircraft that are not routinely and thoroughly dis-insected. There also is a lucrative trade in smuggled pets, so multiple routes of introduction may have been potentially possible. 

Studies done during the summer of 1999 rapidly incriminated the *Cx. pipiens* complex as the likely urban vector [85] and House Sparrows as an important maintenance host [86]; highly infectious American Crows [62] likely were important in virus amplification [87]. Seemingly, the combination of previously introduced urban mosquitoes and birds exploiting periurban habitats in combination with extraordinarily hot weather facilitated the introduction and establishment of WNV. 

**Figure 2:**
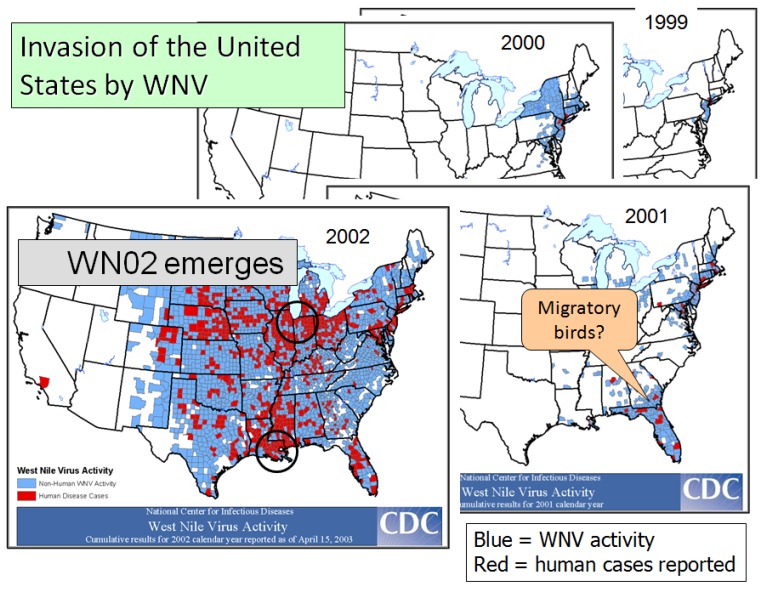
Sequence of maps showing the rapid expansion in the distribution of West Nile virus activity and human cases in the eastern United States from 1999 through 2002. Circles roughly circumscribe epicenters. [http://www.cdc.gov/ncidod/dvbid/westnile/USGS_frame.html].

Despite a large scale adulticiding response, WNV managed to overwinter successfully and then spread slightly in the NE USA during the following summer (Figure 2). During late summer/fall of 2000, the virus seemed to have been carried by southbound migrant birds along the Atlantic flyway, by-passing the midAtlantic states, and becoming established in the Southeast, especially Georgia and Florida, where it amplified during 2001, again in association with hot, dry conditions. However, the peak of the epidemic occurred following years after the virus invaded the west, with epicenters in Chicago and New Orleans in 2002, Colorado in 2003 and Los Angeles in 2004, where *Cx. pipiens, Cx. quinquefasciatus, Cx. tarsalis/Cx. pipiens,* and *Cx. quinquefasciatus,* respectively, were the likely vectors. A hallmark of all these urban epidemics were the huge numbers of American Crows and other bird species dying from infection [22,35] as well as large numbers of horse cases with neuroinvasive disease with high case fatality (http://www.cdc.gov/ncidod/dvbid/westnile/index.htm). Interestingly, like humans, most equine infections seemed to remain subclinical, resulting in high levels of acquired immunity [88]. The equine epidemic was rapidly halted subsequent to 2003 by widespread natural and/or intentional vaccination. 

During 2002, WNV acquired another mutation, E159A in the envelope region of the genome, that rapidly replaced the invading NY99 genotype. This strain, known in the literature as WN02, appeared to enhance *Culex* transmission by allowing the virus to invade the salivary glands sooner after infection than the NY99 strain, especially under warm temperatures [89,90]. Therefore, the virus that invaded the western USA contained both the NS3 mutation causing high viremias and mortality in American Crows and the E mutation that may have enhanced *Culex* transmission, as well as other genetic differences whose functions were not well understood [91]. 

**Figure 3:**
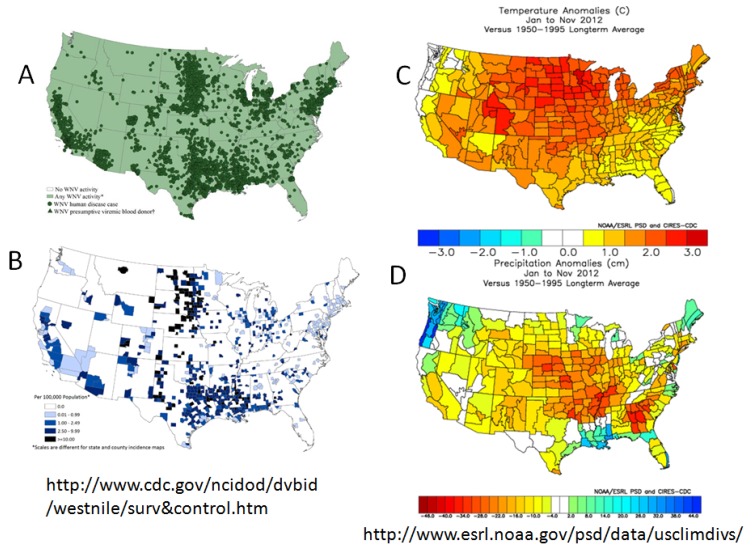
West Nile virus activity and climate analomies in the USA during 2012. West Nile cases: (**A**) Distribution; (**B**) reported case incidence per 100,000; Seasonal climate departures from the 1950–1995 average for the Jan–Nov 2012 period: (**C**) temperature in °C, (**D**) precipitation in cm. (Please confirm the Figure 3 and Figure 4)

Climate variation has been an important factor historically driving SLEV and now WNV transmission to outbreak levels. Typically elevated transmission has been associated with hot, dry weather events [92]. In urban landscapes with a large percentage of impervious groundcover, high rainfall volumes result in rapid run-off that typically ‘flushes-out’ urban waste water systems [93]. Conversely, drought conditions stimulate residence and park landscape irrigation that creates a low volume ‘curb drizzle’, daily refreshing underground systems and catch basins without flushing-out developing larval mosquito populations. In addition, drought conditions may force avian populations into suburban areas where water is more freely available, thereby bringing competent hosts into contact with competent urban vectors. Drought conditions typically are associated with elevated temperatures (http://www.pmel.noaa.gov/tao/elnino/la-nina-story.html), and these conditions are further exacerbated by urban heat island formation [94]. Because mosquitoes are poikilotherms, their body temperature approximates ambient conditions, although *Culex* may behaviorally adjust their temperature by seeking daytime refugia and altering evening activity rhythms [95,96]. In general, warm temperatures increase the rate of larval development and reduce generation time [97], thereby rapidly increasing mosquito population size, and reducing the duration of the gonotrophic [98] and the extrinsic incubation [99] periods. Therefore, during warm temperature anomalies, there frequently are more female mosquitoes, taking more frequent blood meals, thereby increasing host-vector contact and the probability of infection, and infected females are able to transmit virus earlier in adult reproductive life [100] than during cooler seasons. The dramatic shortening of the extrinsic incubation period by warming temperature also compensates for the concurrent decrease in adult survival with warming temperature [101]. The impact of warm temperature has been most noticeable in the US prairie states where the incidence of human infection markedly increases during warm weather anomalies, such as experienced in 2012 (Figure 3). 

Landscape heterogeneity has had a marked impact on the distribution of vector and avian host populations and therefore WNV transmission. Epidemiologically, the greatest number of cases have been detected in urban areas where the most peoplereside, whereas the risk of infection as expressed by case incidence has been highest in rural areas with low human population density, such as the northcentral prairie states. Frequently, the distribution of urban human cases has been delineated spatially by high mortality rates among periurban or urban corvid populations, especially American Crows [102]. All corvids produce elevated viremias and frequently succumb from infection [62,103], and this provides a virus source for effective vector infection, especially when the birds are ill, less mobile and less defensive. In one case control study, residences with dead corvids reported on their property were 19.8 times more likely to also have infected mosquitoes than residences without dead corvids [104]. When the number of dead American Crows around a large communal roost in Los Angeles was delimited spatially using SatScan statistics, the *Cx. quinquefasciatus* minimum infection rate was 8.0 per 1000 within areas circumscribed by dead corvid clusters as opposed to 2.1 per 1000 outside of these clusters; also, only 41% of the human population resided within clusters of dead corvids, but 75% of the laboratory confirmed human cases were reported from within these clusters (incidence within = 5.9, without = 1.3 per 100,000 population, respectively) [105]. As pointed out previously, transmission within these urban areas with reduced avian species richness tends to be more efficient than in rural areas with high species richness, because more blood meals are taken from competent hosts. This was shown in recent surveys of *Culex* blood meal host diversity [67], where blood meal host species richness in urban Los Angeles was half that observed in rural wetlands near Sacramento, where almost every other blood meal came from a different host species, including many that were incompetent hosts for WNV. 

### 4.3. Movement

The rapid dispersal of WNV throughout the New World from NYC to Los Angeles and from Saskatoon, Canada, to Buenos Aires, Argentina, was unexpected, demonstrated the inability of public health interventions to contain an invading vector-borne zoonoses [106], and may have occurred even faster than recorded by surveillance programs, based on an analysis of genetic change in time and space among available isolates [27]. Long distance movement of WNV initially was attributed to migratory birds [28,29,30], and in support, viremic migrants were collected repeatedly during southbound flights from temperate transmission foci [55,58,107]. In contrast, few infected birds were detected during northbound flights from the tropics [107,108], thereby questioning this as a mechanism of rapid east to west movement. In addition, although evidence of WNV presence has been reported repeatedly in the Neotropics and Caribbean [9], foci of human or equine disease have not been detected, indicating limited amplification to levels allowing tangential transmission, and therefore a limited source of virus to be inserted into northbound migrants. Subsequent modeling studies indicated that rapid east-west dispersal could result from post-nesting movement by resident birds and perhaps host-seeking by mosquitoes [109]. During 2004, for example, the movement of WNV from the Los Angeles Basin across the Tehachapi Mountains and into the Central Valley of California occurred after the arrival of Neotropical migrants, but concurrent with post-fledging dispersal by resident birds such as House Finches [110]. Mosquito movement by prevailing storm tracks [111] also would not seem important for east-west WNV dispersal, because weather fronts in NA typically move from west to east and opposite to the dispersal direction of WNV. An unknown was the possible role of commerce moving infected mosquitoes by ground or air transport. The infection of a Los Angeles International Airport employee with WNV in 2002 before the detection of WNV in California by the surveillance program in 2003 may have indicated the long distance transport by an infectious mosquito. 

## 5. Virus Persistence

Establishment of WNV and its subsequent dispersal throughout the New World was contingent upon virus survival over temperate winters, when transmission is interrupted and cold conditions drive the mosquito vectors into diapause or quiescence. Successful overwintering of a tropical African virus in the cold temperate NE USA was not expected, especially since evidence in temperate Europe indicated that the virus did not typically persist between seasons and required re-introduction [112]. Other tropical flaviviruses transmitted by African mosquito vectors such as yellow fever virus historically have been a recurring health problems in the NE USA, but did not overwinter and required annual re-introduction [113]. 

WNV persistence was achieved most likely either by long-term mosquito and/or avian infections. *Culex pipiens,* the vector of WNV in the NYC area, is capable of entering a facultative diapause [36] and was well-adapted to surviving winter conditions. Previous experiments had shown that the F1 progeny of infected *Cx. pipiens* females may become infected vertically [114], thereby providing a mechanism for inserting virus into the next generation without these F1 females taking a blood meal. If these vertically infected females enter diapause/quiescence in response to cool temperature and shortening day length during late summer/fall, then this could provide the mechanism for infecting female mosquitoes collected WNV positive during winter [115,116,117,118]. The following spring when the weather warms, WNV theoretically replicates and these infected mosquitoes become infectious, thereby renewing the transmission cycle. Proof of principal was provided by laboratory experiments, where the F1 progeny of field WNV-infected *Cx. pipiens* females were induced to enter diapause, diapause ‘terminated’ the following ‘spring’, and these F1 female progeny fed on hamsters that became infected [119,120]. Recent field studies provided evidence of frequent vertical transmission by infected females collected during late summer and fall [121]. 

WNV also has been shown to persist as long term infections in avian hosts that survive acute infection. Viral RNA initially was detected in kidney and spleen tissues from multiple species of experimentally infected passerines that were necropsied 6–8 weeks post infection [122]. Subsequent studies showed that RNA could persist for up to 8 months in both experimentally and naturally infected birds [123,124], and that these infections may explain the long term persistence of high titered neutralizing antibody [125]. In agreement, WNV RNA was detected repeatedly in the sera of some birds up to 7 weeks post-infection [126]. In contrast, birds experimentally infected with SLEV rarely established persistent infections and neutralizing antibody titers frequently declined rapidly over time [127,128]. Based on the frequency distributions of qRT-PCR Ct scores from kidney tissues tested for WNV from dead birds submitted by the public, chronic infections with WNV seem to occur frequently in nature, especially among House Finches, House Sparrows and American Robins [129]. The significance of these findings remains uncertain, because virus bound with antibody was not able to infect mosquitoes [84]. However, variation in immune status could play an important role in recrudescence. For example, experimental infection of Rock Doves with WNV showed that some birds developed intermittent viremias over time and that antibody titers increased following detection of infectious virus in sera [130]. In agreement, imperfectly antibody-bound WNV was able to infect a low proportion of susceptible mosquitoes [84], indicating that immunosuppression related to co-infection or the stress of territoriality and nesting during spring could allow some virus to ‘escape’ and infect blood feeding mosquitoes. Other avian pathogens transmitted by *Culex* spp. such as *Plasmodium relictum,* somehow detect seasons and utilize a vernal recrudescence to renew summer transmission [131].

**Figure 4:**
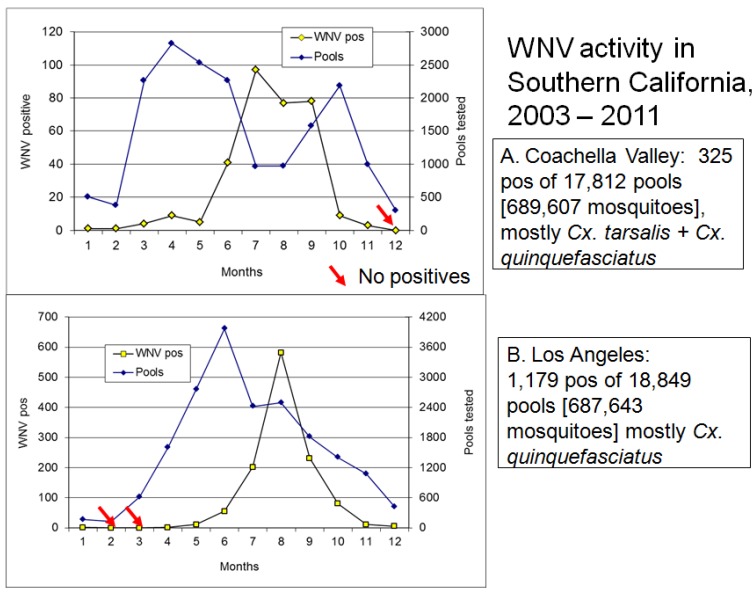
Seasonal activity of West Nile virus in (**A**) Coachella Valley and (**B**) Los Angeles, Southern California summarized from 2003–2011. Graphs plot the total number of mosquito pools tested and positive for WNV per month. Mosquitoes were collected host-seeking at CO_2_ traps or attempting oviposition at gravid female traps and therefore were reproductively active. Red arrows show months when there were no WNV positive pools.

Continued transmission during winter provides an alternate means of virus persistence at both northern and southern latitudes. At temperate latitudes where mosquitoes are inactive throughout the cold winter months, WNV has been detected repeatedly in dead birds. In upstate NY WNV transmission was found to continue at a communal American Crow roost by bird-to-bird transmission, possibly through fecal-oral contamination (feces under the roost were positive), preening (ectoparasites tested positive), and/or cannibalism of carcasses (dead birds were positive) [132]. Bird species positive during winter frequently have been corvids or raptors [133,134] that may have been infected orally, perhaps by eating acute or chronically infected birds. After WNV became established at southern latitudes in the USA, infected birds and mosquitoes were collected throughout the winter months in southern California (Figure 4) and Texas [135], and sentinel chickens in Florida were found to seroconvert to WNV and SLEV throughout the year (http://www.doh.state.fl.us/Environment/medicine/arboviral/surveillance.htm). Although difficult to separate from patterns of progressive vernal warming at increasing northern latitudes, northbound migrants appear to acquire WNV infections during migration at southern temperate latitudes and then transport virus northward during spring. For example, northbound birds collected along the Pacific flyway in the Central Valley of California had higher infection rates than those collected at stop-over points along the Salton Sea near Mexico [108,136]. Similar findings were reported for the Mississippi and Atlantic flyways for WNV [107] and other arboviruses [137,138].

## 6. Outbreaks

### 6.1. Onset of Amplification

The mechanisms and exact timing of the onset of vernal transmission remain obscure, because transmission is inefficient and infrequent, amplification slow, and virus difficult to detect at these low levels. In California, for example, WNV can be detected in an occasional dead bird or mosquito pool in December or January, followed by a period of cessation until May or June [122]. However, it cannot be certain if these occasional positive specimens represent the elongated end of the fall transmission season, winter persistence or transmission, or the onset of new vernal transmission events. For *Culex* mosquitoes that enter winter diapause, the transition in photoperiod after the winter solstice appears to stimulate juvenile hormone production and ovarian development to pre-hosting arrest at ovarian stage I-II, characterized by associated changes in ovarian morphometrics [118,139,140]. This transition from diapause to the arrest stage and the initiation of host-seeking appears to be temperature dependent, occurring shortly and abruptly after the winter solstice in late December at southern latitudes [141], but later and more gradually at more northern latitudes [118,142]. It is not clear, however, if diapause termination actually marks the onset of transmission, because vertically infected and overwintering mosquitoes typically contain minimal amounts of virus that is often difficult to isolate [116,118], indicating that warming and considerable replication is necessary prior to transmission. Viral replication may be exceedingly slow at this time, because few hours/days exceed the 14.3 °C developmental threshold for WNV, resulting in minimal heat accrual until summer hot spells and the increase in nocturnal temperatures [143]. By comparison, *Culex* quiescence is not constrained by photoperiod and winter blood feeding activity may be facilitated by warm periods. However, a concurrent autumnal/winter cessation and winter/vernal initiation of WNV transmission seems to occur at southern latitudes such as Los Angeles, where *Cx. quinquefasciatus* is the primary vector [144], because temperatures are too cold for efficient WNV transmission. In general, transmission in most of the USA seems constrained until the warm summer period, most likely due to the thermodynamics of transmission efficiency described above. Although the slopes of the amplification curve was generally similar [Figure 4], peak activity occurred a month earlier in the hot dry desert of Coachella Valley than in cool coastal Los Angeles. 

Alternatively and coincidental with the initial detection of WNV during May/June, virus may persist in avian hosts and then recrudesce in association with the nesting season. Temperatures at this time are usually sufficient for virus replication in the mosquito host, thereby enabling the possibility of transmission and resulting amplification. However, as discussed previously, additional research is needed, because there are no data to indicate that infections in previously infected birds ever recrudesce and develop viremias sufficient to infect host-seeking *Culex* [126]. 

The level of virus activity during the previous transmission season seems to dictate the probability of viral amplification to outbreak levels. During the invasion phase of the on-going epidemic, WNV typically assumed a three year cycle, with quiet invasion during year one, explosive amplification to outbreak levels during year two, and subsidence during year three [21]. The frequency with which this pattern has been repeated led some investigators to suspect that WNV was actually introduced into NYC in 1998, but was not recognized until 1999. Subsidence during year 3 actually may commence during outbreak year 2 due to the accrual of herd immunity in maintenance host populations such as House Finches and House Sparrows and the depopulation of amplification hosts such as American Crows [34,35,144]. In Los Angeles, for example, the termination of the 2004 outbreak appeared to commence during September 2004, soon after antibody prevalence in peridomestic passerines collectively exceeded 25% [144]. Subsequent Los Angeles outbreaks during 2008 and 2011 followed periods when avian ‘flock’ (aka herd) immunity decreased to less than 10% [68]. In agreement, the large almost nationwide resurgent WNV outbreak during 2012, with the epicenter in Dallas, TX, followed several years of minimal virus activity that undoubtedly allowed flock immunity to be diluted by recruitment and the natural turnover rate of immune birds. Using genetic methods, this subsidence period optimistically was heralded as the waning of the current epidemic [145]. The size and immunity level within competent avian host populations is especially critical during early spring, because at this time *Culex* feed almost exclusively on passerine birds [66,77,146,147] that were potentially exposed to virus during the previous season. In addition, immune parental birds transfer immunity to nestlings through the egg further preventing or at least delaying transmission. So even though host selection favors feeding on competent hosts during the spring period, low flock immunity levels seem critical for efficient virus transmission and amplification [68]. Delay until naïve nestlings are available seems to retard amplification until summer thereby inhibiting large outbreak generation [148,149]. 

Climate variation is also critical for vernal amplification. Warm winters and early termination of mosquito overwintering seem to lead to early season virus amplification. As described previously, with warmer temperatures, there are more vectors, biting more frequently due to shorter gonotrophic periods, potentially transmitting virus earlier in adult life, leading to rapid amplification of virus to outbreak levels earlier during the transmission season than during cooler temperatures. This results in more frequent tangential transmission of virus to humans, especially as vectors shift avian blood feeding patterns to feed more frequently on mammals [77,146,150]. Anthropogenic factors undoubtedly influence transmission during these outbreaks. Summer heat waves contribute to tangential transmission by altering human dress (less clothing worn when it is hot exposing more skin surface to mosquitoes) and behavior (tendency to postpone physical activities until after sunset when vectors are active). Often in summer manual labor associated with agriculture and other outdoor activities are shifted to after sunset and during the host-seeking period of the primary vector species, such as *Cx. tarsalis* and *Cx. quinquefasciatus* [96,151,152]. In contrast, television viewing and air conditioning combine to keep people indoors behind closed windows and thereby may serve as important protective measures limiting host-vector contact and infection [153]. However, in maritime or northern areas with infrequent warm spells, few homes have air conditioning and people therefore may spend more time with open windows or outdoors during hot weather, thereby increasing vector contact.

### 6.2. Economics and Heath Priorities Impact Transmission Dynamics

The economic crisis in US real estate starting in 2006 led to marked increases in home abandonment during 2007–2008. In Bakersfield, CA, for example, there was a 300% increase in the notice of delinquency from the 3^rd^ quarter of 2006 until the 3^rd^ quarter of 2007, and this increase was associated with a comparable and concurrent increase in the number of human cases of WNV, attributed to an undetected increase in the numbers of abandoned and unmaintained swimming pools [154]. Interestingly, this altered suburban/urban landscape dotted with large-sized larval habitats was exploited by the rural vector, *Cx. tarsalis*, as well as the urban vector, *Cx. quinquefasciatus*. The extent of this problem was revealed subsequently using aerial photography and satellite imagery [155], triggering targeted intervention. 

**Figure 5:**
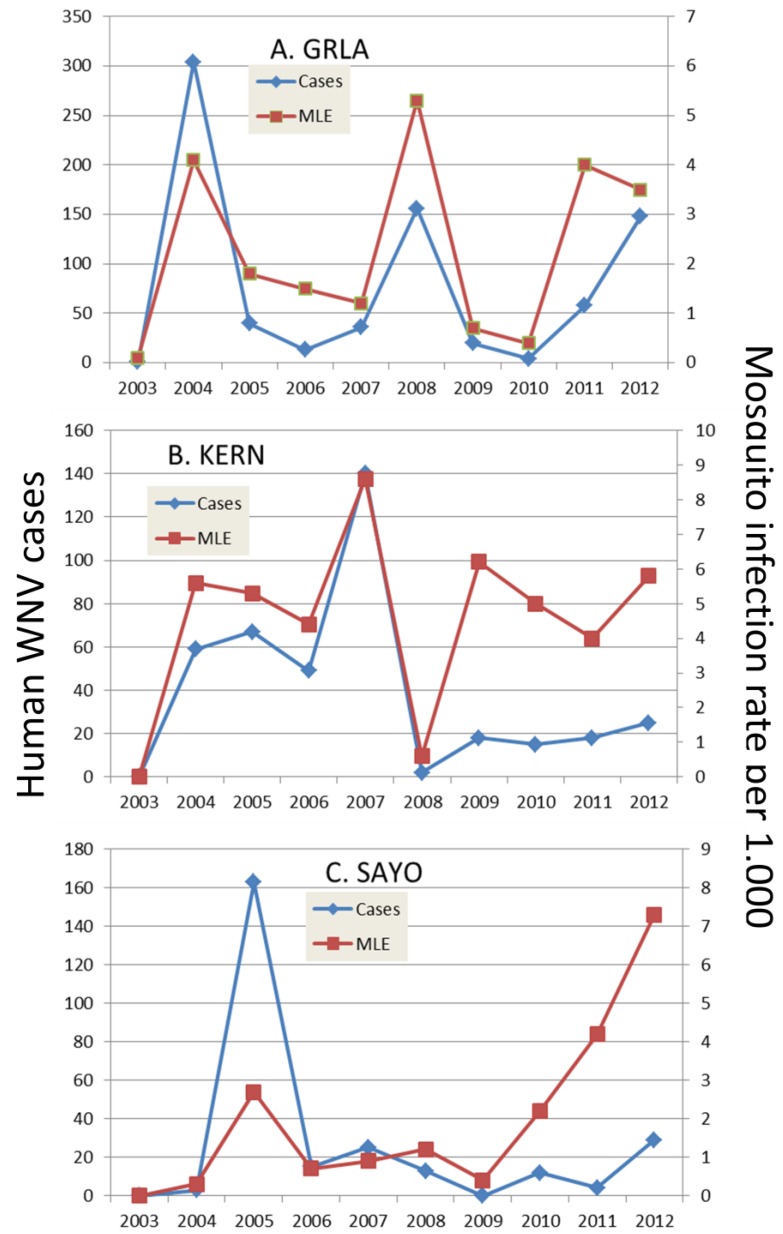
Annual number of human cases and mosquito infection rate per 1000 tested from 2003–2012 for the (**A**) Greater Los Angeles County Vector Control District [GRLA]; (**B**) Kern Mosquito and Vector Control District [KERN] and (**C**) Sacramento-Yolo Mosquito and Vector Control District [SAYO]. Mosquito infection rate was calculated by the maximum likelihood estimate. (confirm the quality of the figure)

The extent and type of mosquito control also may impact outbreak evolution and patterns. In California, most mosquito control districts employ surveillance directed larval treatments, thereby continually pressuring vector populations and reducing adult abundance. Although these methods and improved water management seemed to have eliminated WEEV and SLEV, WNV has remained a public health problem, since its introduction during 2003. Different agencies in different areas of the state respond to surveillance data differently, producing different patterns of virus recurrence and human infection (Figure 5). In Los Angeles, for example, aerial adulticide applications are almost impossible, because of the number of large airports and complex air traffic control issues, and ground applications are complicated by traffic problems. Here, districts respond by public education and enhanced larvicide applications, but these activities seem to allow avian flock immunity to increase and perhaps preclude amplification during years immediately subsequent to outbreaks [68]. In contrast, in Sacramento, the mosquito control program responds rapidly to escalating risk by extending surveillance to delineate the spatial extent of transmission and then by focused aerial adulticide applications to interrupt transmission. These applications seem effective in reducing transmission to humans [156,157] and perhaps to avian hosts as well, thereby precluding elevated flock immunity and allowing effective virus amplification during subsequent years. A similar pattern was observed in Bakersfield, but there the bifurcation of mosquito infection rates and human cases was related to rapid ground adulticide response to surveillance data as well as an intensive program to locate and treat unmaintained swimming pools. These activities seem to have lowered mosquito abundance and the probability of human infection, but have not altered mosquito infection rates.

Elsewhere in the USA, mosquito control programs frequently are a part of local health departments, and therefore focus and funding is often diverted by complex and changing health priorities. Here, outbreak response may be delayed, because skeletal surveillance may not recognize the intensity of amplification and limited resources are available for prompt intervention. Emergency control delayed until a marked increase in human disease incidence is recognized, typically is too late to protect much of the public as well as limit avian infection. The following season surveillance and control typically attracts more fiscal support, but with elevated avian immunity and enhanced control, repeat outbreaks rarely ensue [21]. Again, with waning transmission and health impact, resources become diverted and avian populations recover, setting the stage for subsequent outbreak transmission, similar to as observed in Dallas, TX, and elsewhere during 2012. Continued enzootic surveillance remains the only method of detecting amplification and directing timely intervention to protect the public health. 

## 7. A look to the Future

Host-vector-virus transmission systems are dynamic and typically evolve over time, and this seems to be occurring within the WNV-transmission system. As indicated, the invading virus acquired and retained at least one significant mutation that may have enhanced vector competence and perhaps transmission dynamics under warm [158], but not necessarily moderate [159] temperatures. In California, experimental infections of House Finches [an important maintenance host] comparing the replicative fitness phenotype of the invading WNV 2003 strain carrying a temperature sensitive allele [160] against WNV isolates from four biomes made in 2007-2008 indicated that the founding strain has been replaced by more competitive strains as the virus invaded areas of California such as the Los Angeles Basin and the Central Valley where amplification in corvid populations was important for transmission (Worwa *et al.*, unpublished). Interestingly, there seems to be little concurrent selection for viral change in replicative fitness in House Finches from the SE deserts or for replication within the vector, *Cx. tarsalis*. In addition, repeated estimates of the ID_50_ (virus dose required to infect 50% of exposed mosquitoes) among field *Culex* populations for the NY99 strain of WNV during the invasion of California [161] indicated that there was minimal concurrent change in the vector competence of *Cx. tarsalis* and *Cx. pipiens* complex field populations and that these changes were not associated with outbreaks of human disease. In contrast, progressive sweeps through susceptible avian species populations seem to have selected for resistant phenotypes. This notion was supported by reduced viremia and mortality among House Sparrows and House Finches during successive experimental infection studies [103,123,126,162] (Worwa et al. unpublished). Among birds submitted by the public during the 2010–2011 seasons, these two species also showed a greater frequency of chronic than acute infections as indicated by qRT-PCR Ct scores at necropsy, indicating the frequent natural survival of acute infection [129]. In addition, American crows have been collected with WNV antibody and some dead crows have shown elevated qRT-PCR Ct scores, perhaps indicating they also survived acute infection and died of other causes when chronically infected [129]. Collectively, these data may indicate adaptive changes in avian populations that could reduce the efficiency of transmission unless offset by increases in the virus’s ability to infect vector populations. However, regardless of these apparent trends, the dramatic resurgence of WNV during 2012 certainly indicated that the WNV-transmission system has remained sufficiently intact throughout much of the USA to support widespread epidemic transmission.

**Figure 6:**
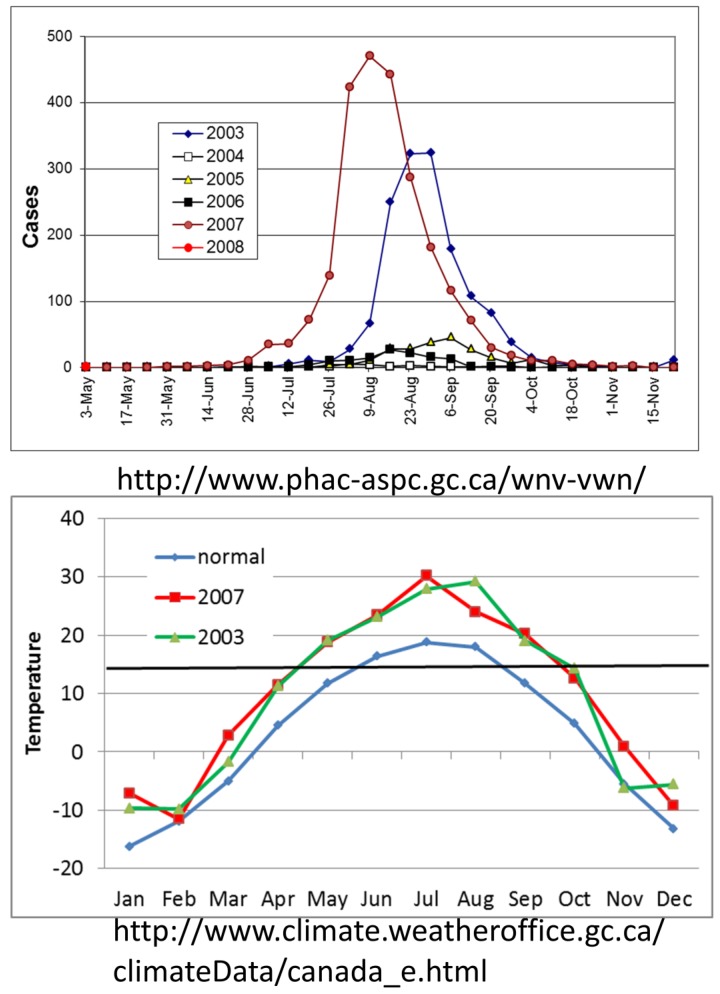
Number of human cases of WNV reported by the Canadian government per year, 2003–2008, and the mean monthly temperature in Regina Saskatchewan in °C for the 50 year average [1950–2000], 2003 and 2007. Horizontal line in bottom panel shows the 14.3 °C threshold for WNV replication.

Regardless of the causal mechanisms and projected rate of temperature change, the earth is becoming warmer, especially at northern latitudes, and these changes will facilitate vector-borne pathogen transmission. Epidemic transmission of WNV in the Canadian prairie provinces with the epicenter in Saskatchewan may serve to illustrate this scenario (Figure 6). Surveillance in Canada recorded epidemics during 2003 and 2007, years when the mean mid-summer temperature in Regina, Saskatchewan, averaged almost 10 °C above the previous 50 year average. This warming dramatically increased the duration of the transmission season when temperatures were above the 14.3 °C replicative threshold for WNV [99] from 3 months on average to 5 and 6 months, respectively, during the 2003 and 2007 epidemic years. Although less dramatic, similar warming trends would be expected to elongate the transmission season and allow efficient transmission in cooler maritime habitats along the cool US West Coast or at higher elevations. In California, the infection of Steller’s Jays with WNV in the Lake Tahoe area at >1900 m [163] was unexpected, but may serve to illustrate the impact of warming trends on the distribution of transmission. 

At present, options for public health intervention appear limited to surveillance directed prevention or emergency responses by organized mosquito control agencies. Surveillance is needed to monitor WNV activity levels within the basic bird-*Culex* transmission cycle to anticipate when and where tangential transmission to humans is likely to occur. The ‘fiscal cliff’, the ‘sequester’ and related national financial problems have combined to seriously alter the US budget devoted to arbovirus surveillance at the national and state levels and thereby direct timely control. WNV has seriously impacted human, animal and wildlife health and cost the US hundreds of millions of dollars in medical costs and emergency intervention. The virus is now firmly established throughout the continental US, remains capable of widespread resurgence as seen in 2012, and most likely will continue to recrudesce whenever environmental conditions support efficient transmission. However, WNV will most assuredly not be the last invasive or re-emerging arbovirus that public and veterinary health agencies have to cope with. This is especially problematic, because most current surveillance systems target laboratory testing specifically for WNV. Although this allows for high throughput diagnostics useful for intervention decision support, these programs will not find what they are not looking for—*i.e.*, other viruses. As with WNV, the establishment of other exotic mosquito vectors, such as *Aedes aegypti* in the Central Valley of California and *Aedes albopictus* throughout the Eastern USA and now Los Angeles elevate the risk for the successful introduction of associated viruses, such as dengue or chikungunya, that have resurged globally and have been repeatedly introduced by travelers within the US [164]. Currently, improved housing and an ‘indoor lifestyle’ seem to be sufficient to preclude autochthonous transmission and establishment [165], but the changes necessary to ’tip’ this balance in favor of these and other viruses are unknown. 
